# A global epidemic serotype 14 *Streptococcus pneumoniae* switching to non-vaccine types

**DOI:** 10.1128/spectrum.03151-24

**Published:** 2025-03-31

**Authors:** Jinglin Yue, Lei Chen, Tingzhu Yao, Pengcheng Du, Chaoyang Gu, Hengkun Wei, Kai Han, Chengbo Rong, Chenchen Wang, Qin Zhang, Chen Chen, Jingyuan Liu, Mingxi Hua

**Affiliations:** 1 Biomedical Innovation Center, Beijing Shijitan Hospital, Capital Medical University 117968 , Beijing, China; 2 Beijing Key Laboratory for Therapeutic Cancer Vaccines, Beijing, China; 3 Children's Hospital of Fudan University at Xiamen 669385 , Xiamen, Fujian, China; 4 Medical Research Center, Beijing Institute of Respiratory Medicine and Beijing Chao-Yang Hospital, Capital Medical University, Beijing, China; 5 Department of Critical Care Medicine, Beijing Ditan Hospital, Capital Medical University 12638 , Beijing, China; Tainan Hospital, Ministry of Health and Welfare, Tainan, Taiwan

**Keywords:** *Streptococcus pneumoniae*, serotype switch, vaccine type, non-vaccine type, antimicrobial resistance

## Abstract

*Streptococcus pneumoniae* infection is a leading cause of bacterial pneumonia worldwide, contributing to both invasive pneumococcal diseases and non-invasive diseases. Following the introduction of pneumococcal conjugate vaccines (PCV7, PCV10, and PCV13), a significant decrease in the prevalence of vaccine-type pneumococci has been observed, while the prevalence of non-vaccine-type (NVT) pneumococci has increased. This shift has been evident in both disease incidence and nasopharyngeal carriage. Serotype switching refers to the alteration or exchange of the serotype-related *cps* gene locus that has been flagged globally as serotypes of concern. In this study, we collected 166 *S*. *pneumoniae* clinical isolates and analyzed the serotypes of isolates to evaluate vaccine effectiveness. Additionally, we identified multidrug-resistant NVT *S. pneumoniae* isolates that recently emerged within the epidemic CC876 complex, a pandemic serotype 14 clone. More importantly, we identified a serotype 15B/ST4749 strain, which might have been generated through a serotype-switch event from serotype 14/ST4749. Mapping the genomic recombination sites within the serotype 15B/ST4749 progeny revealed a donated fragment encompassing *cps, pbp1a,* and additional key factors, including the DNA uptake-related gene *comC*. More importantly, as a strain that is already prevalent, the novel NVT strain possesses the potential to become a global pandemic strain.

**IMPORTANCE:**

The study employed high-throughput sequencing to analyze *Streptococcus pneumoniae* isolates from Xiamen Children’s Hospital, China, to evaluate vaccine effectiveness in light of serotype changes and documented the occurrence of a serotype switch (from 14 to 15B) between vaccine-type and non-vaccine-type serotypes in the ST4749 strain. This observation indicates a genomic recombination and adaptive response of *S. pneumoniae* under selective pressure exerted by vaccination, offering novel insights into strategies for the prevention and control of *S. pneumoniae* and the optimization of vaccine deployment.

## INTRODUCTION

*Streptococcus pneumoniae* infection is a leading cause of bacterial pneumonia worldwide (1), significantly contributing to the global burden of life-threatening invasive pneumococcal diseases (IPDs), such as meningitis and sepsis, and non-invasive diseases (non-IPDs), including sinusitis and otitis media, particularly among young children and the elderly (2–4). Pneumococcal conjugate vaccines (PCVs) have demonstrated effectiveness in preventing invasive pneumococcal diseases, which are a significant health threat to children (5, 6). However, PCVs are not currently part of China’s national immunization program for young children, leading to low vaccination rates and minimal impact on the burden of pneumococcal infections. Specifically, the serotype coverage rates of PCV10, PCV13, and PPSV23 in China range from 20% to 68% in recent studies (7).

Serotype is a crucial determinant of the invasiveness, prevalence, and vaccine targeting of *S. pneumoniae* (8, 9). Following the introduction of the seven-valent pneumococcal conjugate vaccine (PCV7: 4, 14, 18C, 19F, 23F, 6B, and 9V), PCV10 (which adds serotypes 1, 5, and 7F), and PCV13 (which includes serotypes 3, 19A, and 6A), a decline in vaccine-type (VT) pneumococci and an increase in non-vaccine-type (NVT) pneumococci have been observed in both disease incidence and nasopharyngeal carriage (10–12). This phenomenon, known as “serotype replacement (13, 14),” involves the expansion of preexisting NVT strains, while “serotype switching (15, 16)” refers to the alteration or exchange of the serotype-related gene locus. Although non-vaccine serotypes were initially thought to have lower infection potential, and the overall incidence of invasive pneumococcal disease has decreased following the introduction of PCV, serotype replacement remains a significant concern (17). For instance, serotypes 15A and 23B have been flagged globally as serotypes of concern (18–21).

The serotype of *S. pneumoniae* is primarily determined by its polysaccharide capsule, encoded by the *cps* locus. Comprehensive genetic sequencing has revealed that the *cps* locus contains nearly 2,000 coding sequences. These genes can be categorized into three main groups: the first group, positioned upstream of the locus, includes *wzg*, *wzh*, *wzd*, and *wze*, which are conserved across nearly all serotypes (22); the second group consists of serotype-specific genes, such as glycosyltransferases and acetyltransferases, which define their unique serotypes (23); and the third group comprises various sugar synthesis genes critical for capsule production, including rhamnose genes (24). Comparative analysis indicates that recombination is a pivotal factor driving serotype switching, especially following the introduction of PCVs (15). Therefore, sustained surveillance is essential to provide critical epidemiological insights reflecting the dynamic landscape of circulating pneumococcal strains, enabling effective prevention and treatment strategies.

Overall, the most common strains are associated with vaccine serotypes 19F, 19A, 6B, 6A, 23F, and 14, with serotype 14 being the predominant cause of IPDs (25–27). In other countries, serotype 14 strains were globally predominant prior to the introduction of PCVs. However, their prevalence has declined following the nationwide implementation of these vaccines (28). Distinctly, serotype 14/CC876 clones were the dominant serotype in China but presented limited vaccine responses (29). What is more, the CC876 strain of *Streptococcus pneumoniae* exhibits high genomic plasticity (30); however, the specific genomic characteristics have not yet been clearly defined.

In this study, we identified multidrug-resistant NVT *S. pneumoniae* isolates that recently emerged within the epidemic CC876 complex. Based on genomic analysis, we identified a novel serotype 15B/ST4749 strain, which may have originated from a serotype-switch event involving serotype 14/ST4749. Mapping the genomic recombination sites within the serotype 15B/ST4749 progeny revealed a donated fragment encompassing *cps, pbp1a,* and additional key factors, including the DNA uptake-related gene *comC*. More importantly, while the ST4749 strains belonged to the epidemic CC876 complex, the novel NVT strain has the potential to become a global pandemic strain.

## MATERIALS AND METHODS

### Clinical surveillance, bacterial isolation, and antibiotic resistance identification

To investigate the genomic characteristics of *S. pneumoniae* isolates causing infections in children, we conducted a retrospective study in a southern coastal city in China. A total of 166 clinical isolates from patients with *S. pneumoniae* infection were included in this study and obtained through culture on Columbia blood agar base. Antibiotic resistance profiles were identified using the VITEK 2 system, according to the manufacturer’s instructions and the Clinical and Laboratory Standards Institute 2021 standard (31).

### Total DNA extraction and whole-genome sequencing

DNA was extracted using the Genomic DNA Kit (Qiagen, USA), and libraries were prepared with the NEBNext Ultra DNA Library Prep Kit for Illumina (NEB, USA). Paired-end reads of 150 bp were generated on the Illumina NovaSeq 6000 platform, resulting in whole-genome sequencing of 166 *S. pneumoniae* strains. Clean read data were assessed using FastQC (32), and assembly was performed using SPAdes (33) software. Gene prediction and annotation were conducted with Prokka (34).

### Phylogenetic analysis

The assembled genomes were mapped to the complete genome sequence of *S. pneumoniae* Taiwan 19F-14 (accession number: GCA_000019025.1) (35), and single nucleotide polymorphisms (SNPs) were identified using Snippy software (version 4.6.0, https://github.com/tseemann/snippy). Core SNPs from each strain were utilized to infer a maximum likelihood phylogeny using RAxML (36) (version 8.2.8) with the GTR + Gamma rate model. The phylogenetic tree was visualized by the iTOL (version 6) (37) online tool.

### MLST and serotype analysis

Multilocus sequence typing (MLST) was performed using the MLST database (https://pubmlst.org/). Genome sequences were compared with the nucleotide sequences of housekeeping genes (*aroE, gdh, gki, recP, spi, xpt,* and *ddl*) in the MLST database to determine allele counts and assign STs. MLST profiles were characterized using the MLST pipeline with default parameters, and minimum spanning trees were constructed using Phyloviz (version 2.0) (38). Serotypes were identified by comparing sequenced reads against a database containing key genes that determine serotypes within *cps* gene clusters using SeroBA (39).

### Detection of AMR genes, VF genes, and insertion sequences

Antimicrobial resistance (AMR) genes, insertion sequences, and virulence genes were identified by comparing sequences with databases, including Resfinder (40), VFDB (41), and ISfinder (42), respectively, using BLAST (43). The number of AMR genes in VT and NVT samples was analyzed using the Student’s *t*-test to assess the significance of differences between the groups.

### Recombination detection

The genomes of strains S98, S115, S179, S132, and S194 were screened for recombinational blocks with a cutoff of 100 SNPs using Gubbins (44) software. The genomic structures flanking the *cps* region of these strains were analyzed based on a Perl script. The PROKSEE server (https://proksee.ca/) was employed to generate high-quality genome maps of strains S253 and S194.

## RESULTS

### Patient enrollment and sample collection

We conducted a retrospective study to investigate the characteristics of *S. pneumoniae* infection in children under 5 years old in Xiamen, China (Fig. S1). A total of 166 unique *S. pneumoniae* strains were isolated from 166 children with *S. pneumoniae* infection, with an average age of 28.39 months (range: 1–73 months); 90 were male and 76 were female. Age group distribution included 39 patients (23.5%) under 1 year, 43 (25.9%) aged 1–2 years, and 84 (50.6%) over 2 years. The most common clinical diagnosis was pneumonia due to respiratory tract infection (146 cases). Other diagnoses included myelitis (one case), otitis media (three cases), laryngitis (one case), sepsis (one case), convulsion (four cases), infectious fever (seven cases), bronchiectasis (one case), acute gastroenteritis (one case), and acute tonsillitis (one case). Most specimens (158/166, 95.2%) were collected from the respiratory tract, comprising 155 sputum samples, 2 throat swabs, and 1 bronchoalveolar lavage fluid sample. Among the remaining eight strains, five were isolated from blood, two from pus, and one from cerebrospinal fluid (Table 1; Dataset S1).

**TABLE 1:** Demographic and clinical characteristics of 166 patients with *Streptococcus pneumoniae* infection enrolled in this study

	Age group (years)			
	0–1	1–2	>2	Overall
	(*N* = 39)	(*N* = 43)	(*N* = 84)	(*N* = 166)
Sex				
Female	13 (33.3%)	18 (41.9%)	45 (53.6%)	76 (45.8%)
Male	26 (66.7%)	25 (58.1%)	39 (46.4%)	90 (54.2%)
Age (months)				
Mean (SD)	8.44 (3.16)	17.4 (3.39)	43.3 (9.17)	28.4 (16.9)
Median (min, max)	9.00 (1.00, 12.0)	18.0 (13.0, 24.0)	43.0 (25.0, 73.0)	25.0 (1.00, 73.0)
Clinical diagnosis				
Respiratory tract infection	33 (84.6%)	39 (90.7%)	74 (88.1%)	146 (88.0%)
Sepsis	1 (2.6%)	0 (0%)	0 (0%)	1 (0.6%)
Otitis media	0 (0%)	2 (4.7%)	1 (1.2%)	3 (1.8%)
Laryngitis	0 (0%)	1 (2.3%)	0 (0%)	1 (0.6%)
Myelitis	0 (0%)	1 (2.3%)	0 (0%)	1 (0.6%)
Convulsion	3 (7.7%)	0 (0%)	1 (1.2%)	4 (2.4%)
Infectious fever	1 (2.6%)	0 (0%)	6 (7.1%)	7 (4.2%)
Bronchiectasis	1 (2.6%)	0 (0%)	0 (0%)	1 (0.6%)
Acute gastroenteritis	0 (0%)	0 (0%)	1 (1.2%)	1 (0.6%)
Acute tonsillitis	0 (0%)	0 (0%)	1 (1.2%)	1 (0.6%)
Clinical specimen type				
Blood	1 (2.6%)	1 (2.3%)	3 (3.6%)	5 (3.0%)
Cerebrospinal fluid	1 (2.6%)	0 (0%)	0 (0%)	1 (0.6%)
Sputum	35 (89.7%)	41 (95.3%)	79 (94.0%)	155 (93.4%)
Throat swab	2 (5.1%)	0 (0%)	0 (0%)	2 (1.2%)
Bronchoalveolar lavage fluid	0 (0%)	0 (0%)	1 (1.2%)	1 (0.6%)
Pus	0 (0%)	1 (2.3%)	1 (1.2%)	2 (1.2%)

### Vaccine-type and non-vaccine-type serotypes isolated were phylogenetically related in *S. pneumoniae*

**Fig 1:**
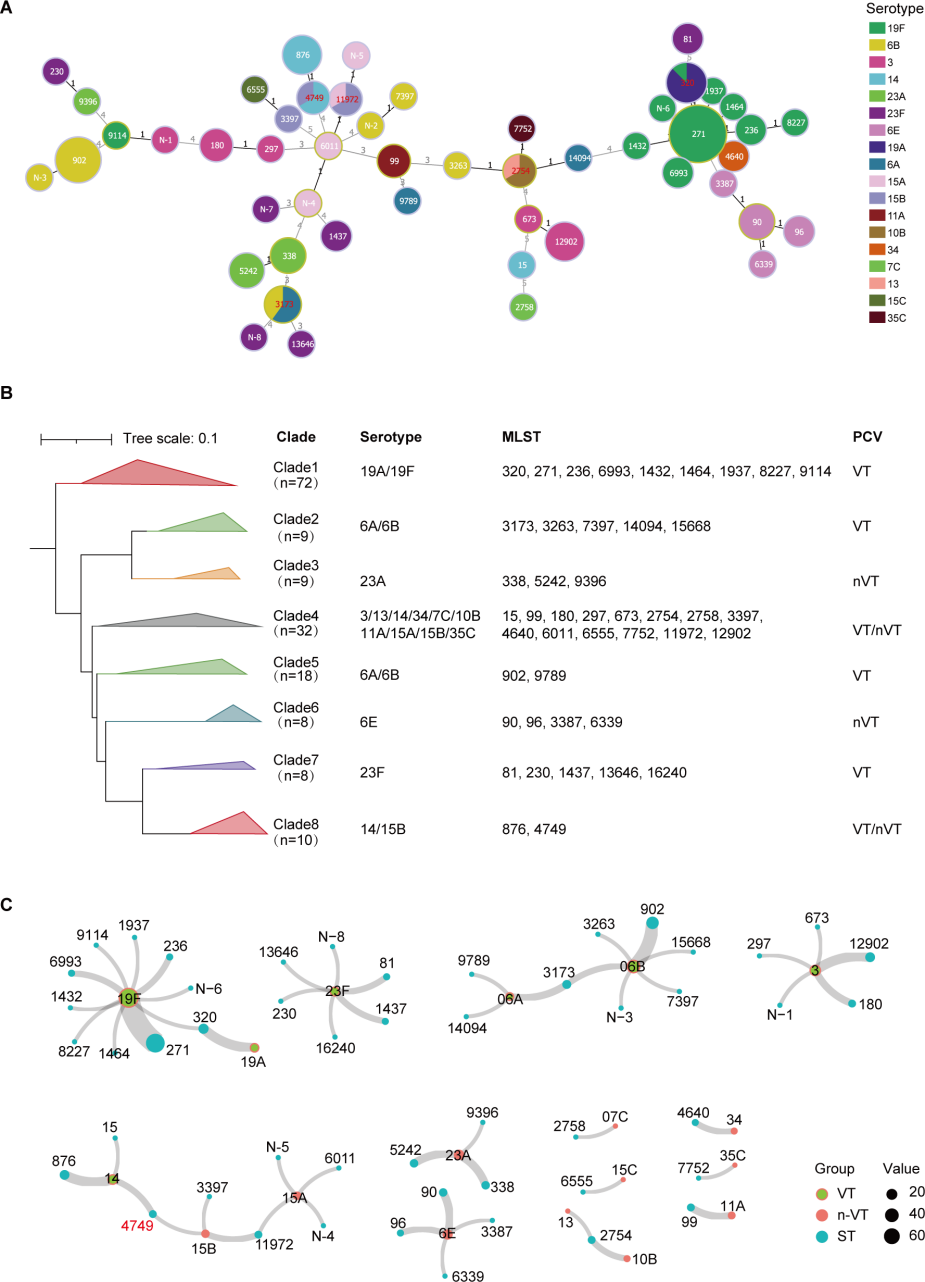
(A) The minimum spanning tree of 166 *Streptococcus pneumoniae* genomes. Color indicates serotype. A minimum spanning tree based on genome sequence type (ST). Each circle represents a single ST. (B) The phylogenetic tree of 166 *Streptococcus pneumoniae* genomes that are classified into eight clades. Color indicates tree clade. (C) The network between serotypes and MLST types of 166 *S. pneumoniae* strains. Point colors represent VT serotypes, n-VT serotypes, and MLST types, while point sizes correspond to the number of strains. Line width indicates the frequency of co-occurrence between a specific serotype and ST within the same strain.

Although the immunization coverage in China is comparatively lower than those observed in European and American nations, vaccination remains a pivotal element in the prophylaxis against pneumococcal infections. We then analyzed the serotype distribution of these *S. pneumoniae* strains according to their *cps* locus. The result showed that all 166 isolates were assigned to 18 serotypes, and 78.3% (130/166) of the isolates belonged to VT serotypes, which were 19F, 19A, 06A, 06B, 3, and 14 (Fig. 1A). Among the NVT serotype strains, 23A (Clade 3) and 6E (Clade 6) were the most common types and accounted for 5.4% (9) and 4.8% (8) of infection cases, respectively, with the remaining serotypes including 15B, 15A, 11A, 34, 10B, 13, 35C, and 7C. (Fig. S2; Table S1).

**Fig 2:**
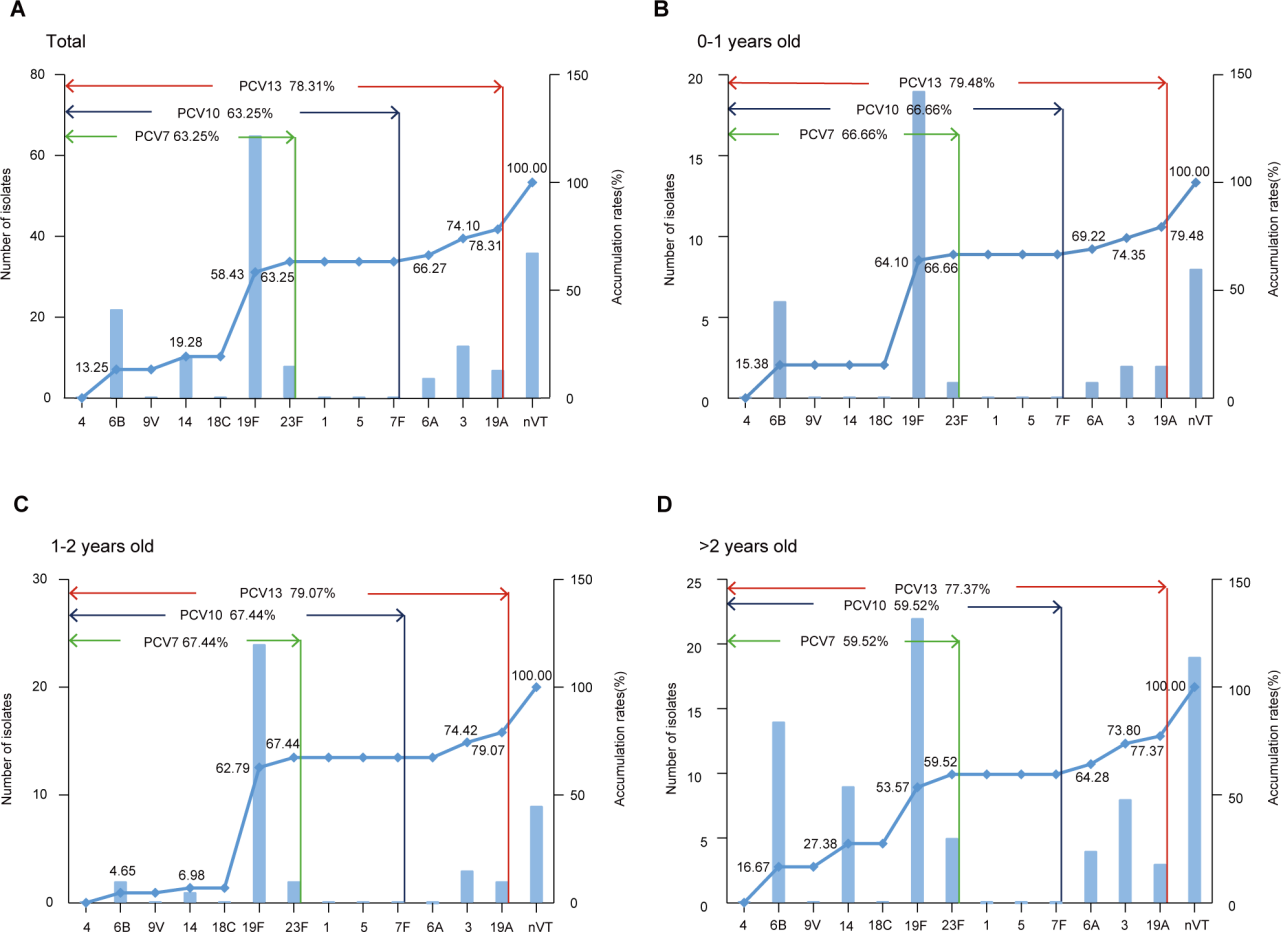
(A) The isolate numbers and accumulation rates for each serotype of *Streptococcus pneumoniae* among all patients. The blue bar plot indicates the number of isolates, while the blue line represents the accumulation rate. The PCV7, PCV10, and PCV13 serotypes are highlighted. (B) The isolate numbers and accumulation rates for each serotype of *Streptococcus pneumoniae* among patients aged 0–1 year. Others are as above. (C) The isolate numbers and accumulation rates for each serotype of *Streptococcus pneumoniae* among patients aged 1–2 years. Others are as above. (D) The isolate numbers and accumulation rates for each serotype of *Streptococcus pneumoniae* among patients aged older than 2 years. Others are as above.

Next, we calculated the coverage rate of different PCVs against the serotypes identified in the above *S. pneumoniae* strains. The coverage rates of PCV7 and PCV13 among hospitalized children were 63.25% and 78.31%, respectively. For patients aged <1 year, 1–2 years, and >2 years, the PCV7 coverage rates were 66.66%, 67.44%, and 59.52%, while the PCV13 coverage rates were 79.48%, 79.07%, and 77.37%, respectively (Fig. 2). This may imply that the inclusion of pneumococcal vaccines in the routine immunization schedule is more beneficial for children at a younger age.

Third, we explored the genetic relationships between VT and NVT strains. We obtained 2, 112, and 148 SNPs, performed phylogenetic analysis, and observed high diversity among the 166 genomes, with eight lineages (Clades 1–8) (Fig. 1B). To understand the epidemic characteristics of *S. pneumoniae*, we identified the MLSTs and serotypes of the 166 strains based on the genomic data. The 166 isolates were assigned to 50 STs (Fig. 1B; Table S1). Consistent with previous studies, ST271 strains of serotype 19F were the predominant type of *S. pneumoniae* and accounted for 39.2% of the infections (65 cases). Furthermore, as shown in Fig. 1, serotype 23A strains were phylogenetically related to serotype 6A/6B strains (Clade 3), and serotype 6E strains were phylogenetically related to serotype 23F and 14 strains (Clade 6). VT and NVT serotype isolates were phylogenetically related in *S. pneumoniae*, which suggests that serotype switching might occur among genetically proximate isolates of *S. pneumoniae*.

### VT to NVT serotype-switch variant of multidrug-resistant *S. pneumoniae* sequence type 4749

**Fig 3:**
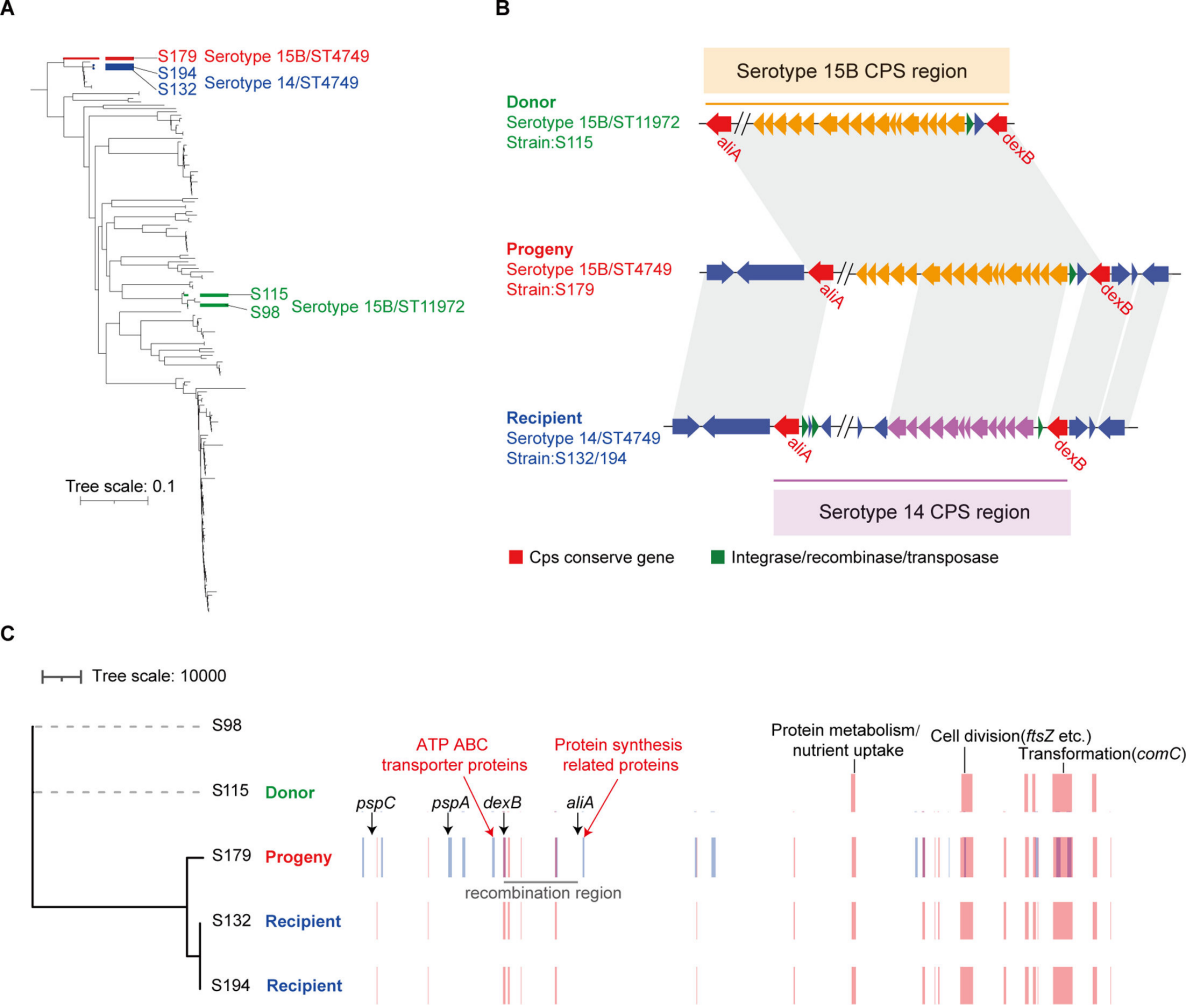
(A) Phylogenetic tree illustrating the progeny serotype 15B/ST4749 isolate S179 (red line), putative recipient serotype 14/ST4749 isolates S132 and S194 (blue line), and putative donor serotype 15B/ST11972 isolate S115 (green line). The scale bar represents the relative branch length. (B) Gene composition of the *cps* region in isolates S115 (donor), S179 (progeny), and S132/194 (recipient). The *aliA* and *dexB* genes (red arrow) were conserved at the end of the *cps* region. Orange arrow indicates serotype 15B *cps* region. Purple arrow indicates serotype 14 *cps* region. (C) Phylogenetic alignment of isolates S98, S115, S179, S132, and S194 and a schematic of recombinant genome fragments, represented by rectangular blocks that were predicted by Gubbins with the parameter of 100 SNPs. Block locations and sizes are relative to the aligned genomes; red blocks represent sites in common isolates, blue blocks represent sites unique to a given isolate, and the gray line represents the serotype-switch fragment that replaced the corresponding *cps*15B region within the recipient ST4749/serotype 14 isolates S132 and S194. The black arrows indicate virulence genes (*pspC* and *pspA*) and *cps*-conserved genes (*dexB* and *aliA*). The red arrows indicate associated proteins surrounding the *cps*15B region, and other proteins in all isolates are highlighted with black lines.

We analyzed the relationship between serotypes and sequence types in *S. pneumoniae*. Our results revealed that 8 of the 18 serotypes were detected inspecific STs, while the remaining 10 serotypes were associated with two or more STs (Table S1). For example, the predominant serotype 19F was distributed across 10 STs: ST236, ST271, ST320, ST1432, ST1464, ST1937, ST6993, ST8227, ST9114, and a new unknown ST. Among STs, ST271 was the dominant type, while ST9114 was phylogenetically distinct from the other nine STs. Although these 10 STs belong to the same serotype 19F, meaning they share similar *cps* locus regions, they are distributed across different phylogenetic lineages. These serotype switches may result from horizontal gene transfer events involving the *cps* locus regions among different ST types of *S. pneumoniae* (Fig. 3A; Table S1).

Additionally, we observed serotype-switch events within strains belonging to the same ST but exhibiting different serotypes. In this study, five serotype-switch events were detected, including switches between 19F and 19A, 6A and 6B, 15A and 15B, 14 and 15B, and 13 and 10B (Table S1). Among them, serotype 14 is a globally prevalent VT strain; however, serotype 15B strain was not protected by vaccines. So, this serotype switch indicates a risk of an epidemic caused by novel NVT *S. pneumoniae* strains (Fig. 1C; Table S1). Thus, we defined this NVT strain as serotype 15B/ST4749 lineage using multi-locus sequence typing and whole-genome sequencing data in this study.

To identify genomic regions contributing to the serotype 15B/ST4749 progeny (strain S179) resulting from recombination, we first identified likely recipient and donor strains in the serotype-switch event. Phylogenetic analysis using 166 genome sequences revealed two serotype 14/ST4749 genomes (strains S132 and S194) that shared genome sequence similarity, with an ANI value of 99.35%, to serotype 15B/ST4749. These two serotype 14/ST4749 strains were the most highly related putative genetic recipients (Fig. 3A). By using BLAST, we identified the likely *cps* region donor, serotype 15B/ST11972 strain S115, which shared about 90% sequence identity to the *cps* region of serotype 15B/ST4749 progeny (*dexB* to *aliA*) (Fig. 3B).

To pinpoint recombination sites, we screened the genomic sequences of strains S179, S115, S132, and S194 using Gubbins software, identifying 17 specific recombinational fragments within S179 (median size: 6,188 bp [range: 1,990–11,344 bp]). Comparative analysis of the genes encoded in the serotype-switch region of the recipient with corresponding regions from the progeny and donor revealed that they uniformly encoded genes involved in protein metabolism, cell division (*ftsZ*), and transformation (*comC*) (Fig. 3C).

### The specific genomic characteristic might be responsible for the serotype switch

**Fig 4:**
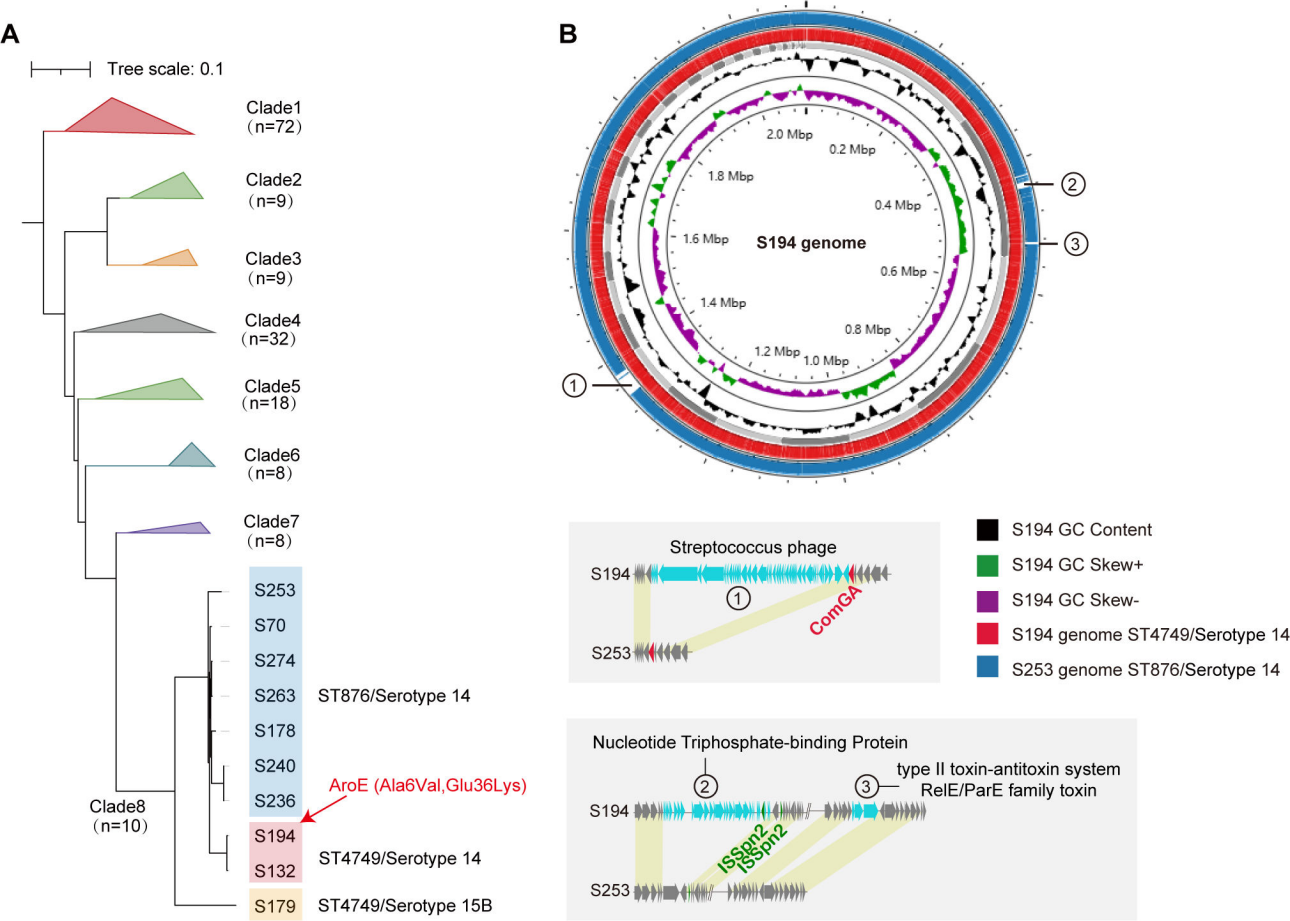
(A) The phylogenetic tree of 166 *Streptococcus pneumoniae* genomes classified into eight clades. Color indicates tree clade. The genomes within Clade 8 were highlighted and marked with ST and serotypes. The AroE allele (Ala6Val and Glu36Lys) was detected exclusively in ST4749 genomes, as shown on the branch. (B) Comparative genomic circle plot of genomes S194 (red circle) and S253 (blue circle), indicating the functions of three regions unique to genome S194. The comparative gene composition of these three unique regions between the S194 and S253 genomes is shown, with cyan arrows denoting genes that are unique to S194.

According to previous studies, serotype 14/ST876 is a specific prevalent strain in China, but we did not observe any serotype-switch events among ST876 strains in clinical isolates. Whereas, in this study, we observed three novel ST4749 strains, which have a single site variant from ST876 strains. As of the current time, extensive whole-genome sequencing data for ST4749 are absent from public databases, thereby constraining our comprehension of its genetic origins. According to the complete genomic analysis, we detected three novel ST4749 strains within clade 8, each exhibiting two missense mutations in the *aroE* gene (Ala6Val and Glu36Lys) compared to ST876 strains (Fig. 4A). The results implied the influence of the housekeeping genes on the serotype of *S. pneumoniae*.

Despite a lower isolation rate compared to ST876 (seven compared to three), we detected a serotype switch in one of the three ST4749 strains. To investigate whether this genomic recombination is affected by the genomic characteristic of the strains, we analyzed the whole-genome sequences of S253 and S194, which were ST876 and ST4749 strains, respectively (Fig. 4B). Compared to S253 (ST876 strain), S194 (ST4749 strain) exhibited more gene numbers (2,145 vs 2,042) with three insertion sequences. The insertion sequences referred to phage, nucleotide triphosphate-binding protein, and type II toxin-antitoxin system RelE/ParE family (Fig. 4C); whether these genes account for the *cps* loci recombination is still to be investigated.

### Antimicrobial resistance features of the isolates and resistance gene carriage

Given the rising antimicrobial resistance rates of *S. pneumoniae*, which severely limit antibiotic options for pediatricians, we analyzed the resistance phenotypes of the 166 collected strains and the distribution of drug-resistant genes. We tested the susceptibility of the 166 strains to 14 antimicrobial agents across 10 classes, including penicillin, cephalosporins (ceftriaxone and cefixime), carbapenems (ertapenem and meropenem), quinolones (ofloxacin, moxifloxacin, and levofloxacin), sulfonamides (sulfamethoxazole), tetracycline, chloramphenicol, macrolides (erythromycin and telithromycin), linezolid, and vancomycin. Resistance profiles were analyzed. The rates of susceptibility, intermediate resistance, and resistance to penicillin in VT *S. pneumoniae* were 8.5%, 16.2%, and 75.4%, respectively. In contrast, the resistance rate for NVT *S. pneumoniae* was 44.44%, significantly lower than that of VT strains. Similar patterns were observed for cephalosporins, carbapenems, quinolones, sulfonamides, and tetracycline, where VT strains exhibited higher resistance rates than NVT strains. Conversely, resistance rates to erythromycin (a macrolide antibiotic) and chloramphenicol were lower in VT compared to NVT strains. Most strains were sensitive to vancomycin, telithromycin, and linezolid, although some strains were not tested (NA in the table) (Table S2).

**Fig 5:**
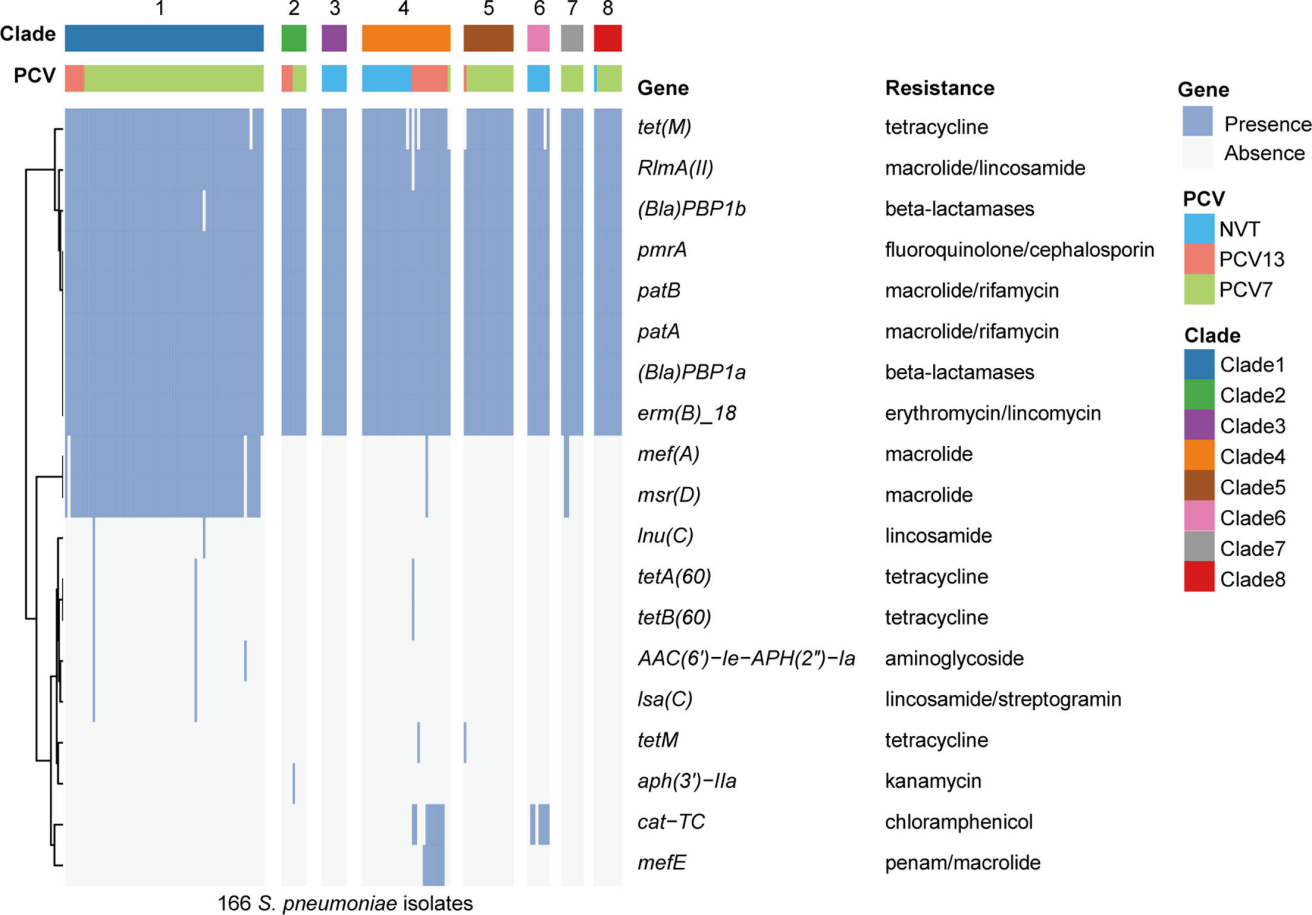
The heatmap shows antibiotic resistance genes for PCV7, PCV13, and NVT in *Streptococcus pneumoniae*. Blue and white blocks indicate gene presence and absence, respectively. Color indicates phylogenetic clade and PCV type.

Recently, the Asian Network for Surveillance of Resistant Pathogens study group performed a prospective surveillance study on serious pneumococcal infections in Asian countries. Antibiotic resistance to β-lactams and macrolides was commonly observed (45). In our study, we analyzed the carriage of drug-resistant genes based on whole genome data. A total of 19 AMR genes were identified, with a broad spectrum of resistance covering nine antibiotic classes (fluoroquinolone, rifamycin, aminoglycoside, beta-lactamases, chloramphenicol, macrolide, streptomycin, trimethoprim, and tetracycline) (Fig. 5). The high AMR gene carriage rates included *tetM* (95.78%) for tetracycline resistance, (*Bla*) *PBP1a* (100%) and (*Bla*) *PBP1a* (99.40%) for beta-lactam resistance, *patA* (100%), *patB* (100%), *erm*(*B*)_*18* (100%), and *RlmA* (*II*) (99.40%) for macrolide resistance, and *pmrA* (100%) for fluoroquinolone resistance, consistent with the observed resistance phenotypes. Besides, there were no differences in AMR genes among the five serotype-switch events (Table S3). However, compared to NVT serotype strains, VT serotype strains generally harbored more AMR genes (9.31 ± 1.19 vs 8.11 ± 0.40, *P* < 0.01), particularly the *mef(A)*, *msr(D)*, *cat-TC*, and *mefE* genes. Among VT serotypes, the 19A/19F serotype strains in Clade 1, as a globally antibiotic-resistant representative clone, specifically carry macrolide resistance genes *mef(A)* and *msr(D)*, which may be associated with the types of antibiotics used in 19A/19F IPD infections.

## DISCUSSION

As the primary cause of bacterial pneumonia, otitis media, meningitis, and septicemia, *Streptococcus pneumoniae* poses a significant global health challenge. In this study, we highlighted that most infection cases are caused by serotypes targeted by vaccine-type *S. pneumoniae* strains, underscoring the critical role of vaccination in preventing pneumococcal pneumonia in children. PCV13 has been significantly effective in reducing the incidence of invasive pneumococcal disease in children. However, the prevalence of non-vaccine serotypes, such as serotypes 15A and 23A, has increased (46). Additionally, in this study, we confirmed that the antimicrobial resistance genes in VT strains are more than NVT strains; nevertheless, the increase in antimicrobial resistance caused by NVT strains requires ongoing surveillance (47).

*S. pneumoniae* is a highly recombinogenic pathogen. While serotype switching driven by recombination at the *cps* locus remains a concern, it highlights the dynamic nature of its genetic evolution (15, 24). In addition, there is limited global evidence of a sustained serotype replacement from 19F to 19A in terms of carriage and disease following the introduction of PCV7 and PCV10 (48–50). Thus, understanding serotype transitions, particularly from VT to NVT serotypes, is crucial for the development of advanced, costlier vaccines to ensure comprehensive protection against *S. pneumoniae*.

Research indicates that the serotype 14/ST876 strain, which was only prevalent in China, exhibits a poor response to vaccines, primarily due to its increased recombination activity at the *cps* locus and the unique mutation patterns in the *wzg* and *lrp* genes. These genetic alterations may impair vaccine recognition and the effectiveness of the immune response, thereby allowing the serotype 14/ST876 strain to evade vaccine protection (29). In this study, we employed whole-genome sequencing to identify a VT to NVT switch: a capsular switching from serotype 14 to serotype 15B in ST4749 strains. ST4749 and ST876 strains differ by only a single locus mutation (AroE: Ala6Val, Glu36Lys), so the novel multi-antimicrobial resistance serotype 15B/ST4749 was possibly derived from an ST876 strain. This observation offers an opportunity to investigate the genomic characteristics influencing *cps* locus recombination in *S. pneumoniae.*

Serotype switching refers to a change in serotype within a single strain, achieved by altering its capsular polysaccharide synthesis genes (15). In contrast, serotype replacement is a population-level phenomenon in which the number of VT strains decreases while the number of NVT strains increases due to external factors (12). Although this study supports the occurrence of serotype switching, it does not fully consider alternative explanations, such as the independent expansion of non-vaccine serotype strains or incomplete vaccine coverage, both of which could also account for the observed pattern. Future studies should include more strains to evaluate these alternative explanations thoroughly and gain a more accurate understanding of the mechanisms behind serotype change from VT to NVT in *S. pneumoniae*. Overall, the spread of recombinant multidrug-resistant pneumococcal clones with NVT serotypes is concerning and highlights the need for rigorous monitoring of serotype and genotype dynamics within pneumococcal populations (51, 52).

## Data Availability

All genome sequences from this study have been deposited in the National Genomics Data Center (https://ngdc.cncb.ac.cn/), under the BioProject accession number PRJCA031327.

## SUPPLEMENTAL MATERIAL

Dataset S1 spectrum.03151-24-s0001.xlsx Clinical information of 166 patients with *Streptococcus pneumoniae* infection enrolled in this study.

Figure S1 spectrum.03151-24-s0002.tif Flow chart of patients enrolled in the study.

Figure S2 spectrum.03151-24-s0003.tif Phylogenetic tree of 166 *Streptococcus pneumoniae* clinical isolates.

Tables S1 to S3 spectrum.03151-24-s0004.docx Description of all supplemental materials and Serotype, ST, and antibiotic resistance information of isolates.

ASM does not own the copyrights to Supplemental Material that may be linked to, or accessed through, an article. The authors have granted ASM a non-exclusive, world-wide license to publish the Supplemental Material files. Please contact the corresponding author directly for reuse.
