# Supplementary figures and images for: A global epidemic serotype 14 Streptococcus pneumoniae switching to non-vaccine types
Source: Microbiol Spectr. 2025 Mar 31;13(5):e03151-24. doi: 10.1128/spectrum.03151-24 (PMC12054029; doi:10.1128/spectrum.03151-24)

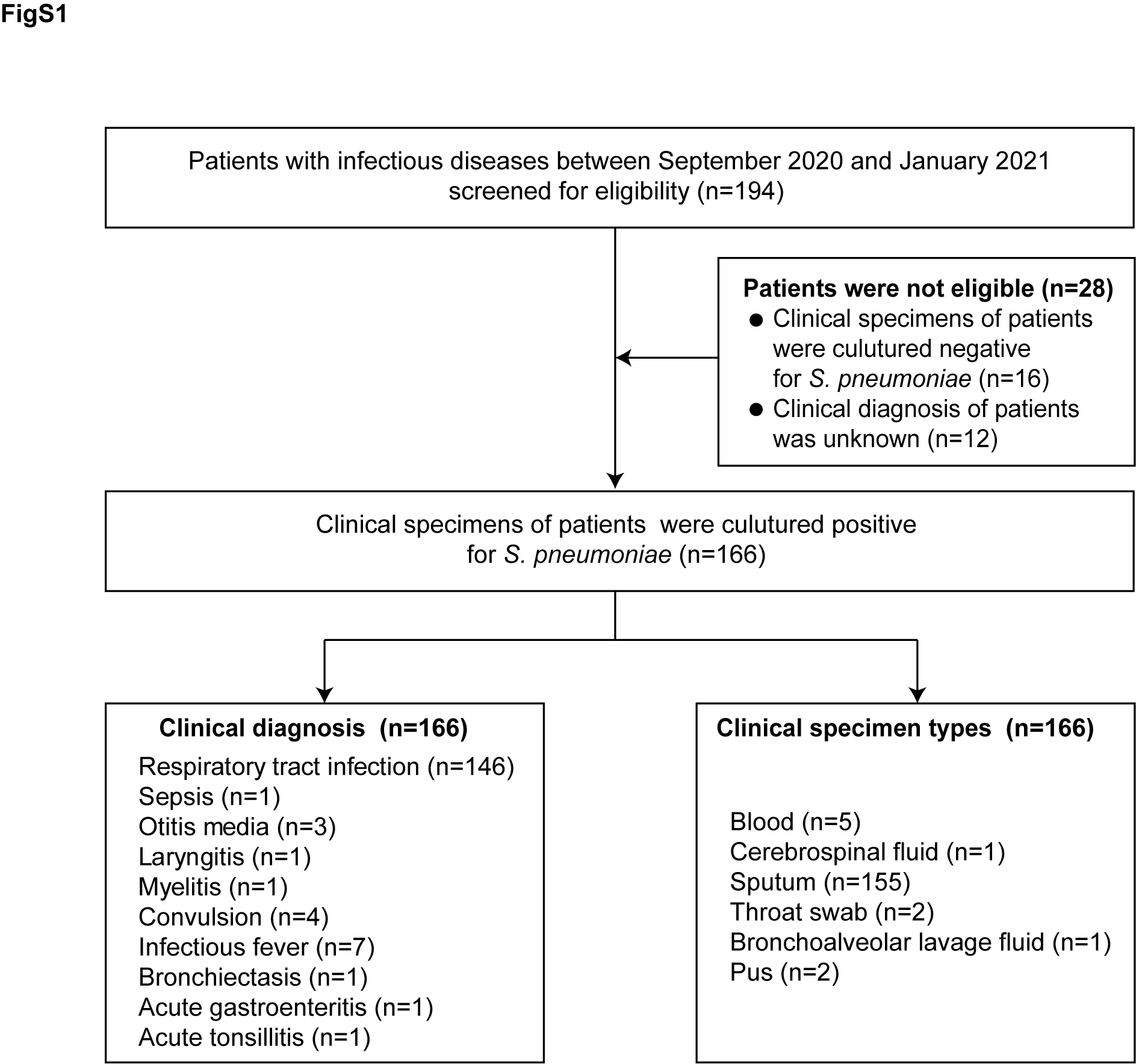

Supplement: Figure S1 — Flow chart of patients enrolled in the study. [file spectrum.03151-24-s0002.tif]

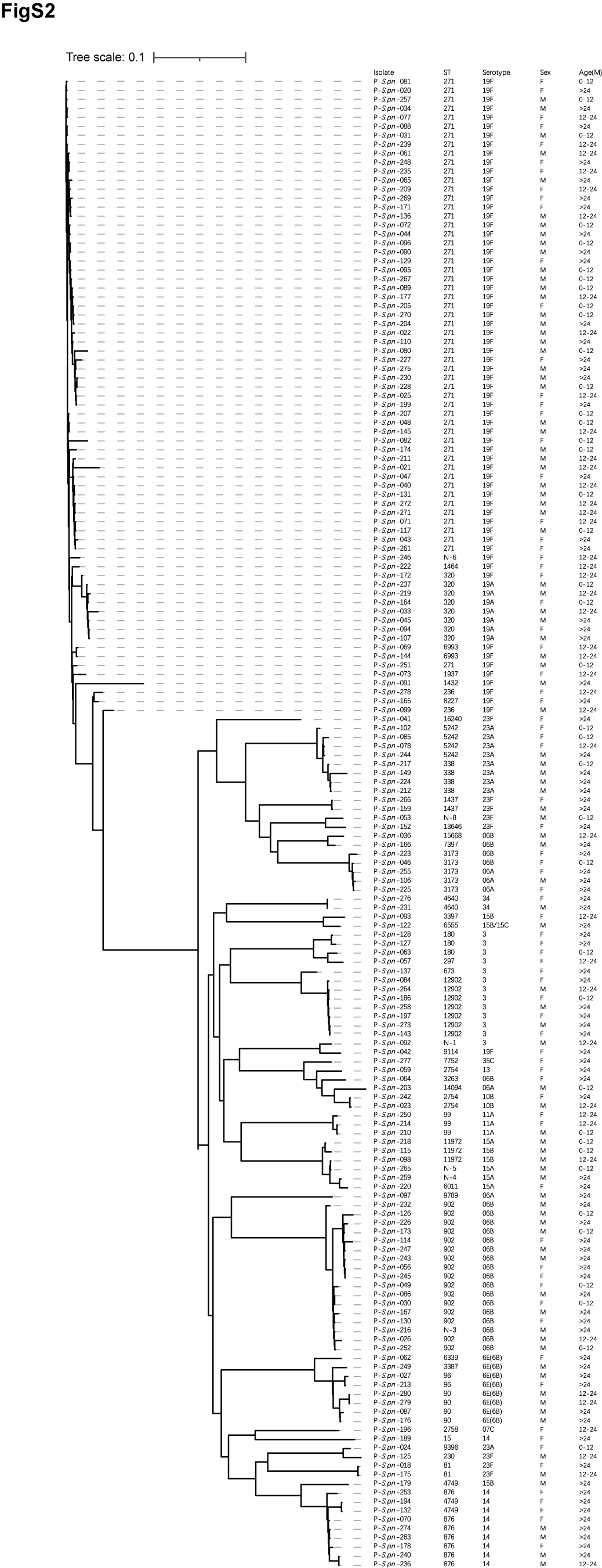

Supplement: Figure S2 — Phylogenetic tree of 166 Streptococcus pneumoniae clinical isolates. [file spectrum.03151-24-s0003.tif]
