# Supplementary material for: A global epidemic serotype 14 Streptococcus pneumoniae switching to non-vaccine types
Source: Microbiol Spectr. 2025 Mar 31;13(5):e03151-24. doi: 10.1128/spectrum.03151-24 (PMC12054029; doi:10.1128/spectrum.03151-24)
Supplement: Tables S1 to S3 — Description of all supplemental materials and Serotype, ST, and antibiotic resistance information of isolates. [file spectrum.03151-24-s0004.docx]

**Supplemental material**

**Figure S1.** Flow chat of patients enrolled in the study.

**Figure S2.** The phylogenetic tree of 166 *Streptococcus pneumoniae* clinical isolates. Isolate id, ST type, serotype, patient sex and patient age of each strain is documented. The N-x of ST represents a new ST type or new allele gene types.

**Table S1.** The distribution of ST/Serotype in 166 *Streptococcus pneumoniae*.

**Table S2.** Resistance phenotypes of VT and NVT strains in *Streptococcus pneumoniae*.

**Table S3.** Antimicrobial resistance genes of strains associated with serotype switches in *Streptococcus pneumoniae*.

**Dataset S1.** Clinical information of 166 patients with *Streptococcus pneumoniae* infection enrolled in this study.

**Table S1. The distribution of ST/Serotype in 166 *Streptococcus pneumoniae***

| Serotype | ST | Genome number |
| --- | --- | --- |
| 6E | 90 | 4 |
|  | 96 | 2 |
|  | 3387 | 1 |
|  | 6339 | 1 |
| 35C | 7752 | 1 |
| 23F | 81 | 2 |
|  | 1437 | 2 |
|  | 230 | 1 |
|  | 13646 | 1 |
|  | 16240 | 1 |
|  | Unknown | 1 |
| 23A | 338 | 4 |
|  | 5242 | 4 |
|  | 9396 | 1 |
| 19F | 271 | 54 |
|  | 236 | 2 |
|  | 6993 | 2 |
|  | 320 | 1 |
|  | 1432 | 1 |
|  | 1464 | 1 |
|  | 1937 | 1 |
|  | 8227 | 1 |
|  | 9114 | 1 |
|  | Unknown | 1 |
| 19A | 320 | 7 |
| 15B | 11972 | 2 |
|  | 3397 | 1 |
|  | 4749 | 1 |
|  | 6555 | 1 |
| 15A | Unknown | 2 |
|  | 6011 | 1 |
|  | 11972 | 1 |
| 11A | 99 | 3 |
| 10B | 2754 | 2 |
| 07C | 2758 | 1 |
| 06B | 902 | 16 |
|  | 3173 | 2 |
|  | 3263 | 1 |
|  | 7397 | 1 |
|  | 15668 | 1 |
|  | Unknown | 1 |
| 06A | 3173 | 3 |
|  | 9789 | 1 |
|  | 14094 | 1 |
| 34 | 4640 | 2 |
| 14 | 876 | 7 |
|  | 4749 | 2 |
|  | 15 | 1 |
| 13 | 2754 | 1 |
| 3 | 12902 | 7 |
|  | 180 | 3 |
|  | 297 | 1 |
|  | 673 | 1 |
|  | Unknown | 1 |

**Table S2. Resistance phenotypes of VT and NVT strains in *Streptococcus pneumoniae***

|  |  | Penicillin | Cephalosporin | | Carbapenem | | | Macrolide | | | Quinolone | | | | Sulfonamides | | Linezolid | Tetracycline | Vancomycin | Chloramphenicol |
| --- | --- | --- | --- | --- | --- | --- | --- | --- | --- | --- | --- | --- | --- | --- | --- | --- | --- | --- | --- | --- |
|  |  | PEN | CTX | CRO | | ETP | MEM | ERY | | TEL | OFX | | MFX | LEV | SXT | | LNZ | TCY | VAN | CHL |
| **VT** | S | 8.5% | 89.2% | 90.0% | | 92.3% | 91.5% | | 0.8% | 84.6% | | 90.8% | 100% | 98.5% | | 10.0% | 93.1% | 6.2% | 100% | 92.3% |
|  | I | 16.2% | 3.1% | 2.3% | | 1.5% | 2.3% | | 0.0% | 0.8% | | 2.3% | 0.0% | 0.8% | | 23.1% | 0.0% | 0.0% | 0.0% | 0.0% |
|  | R | 75.4% | 6.2% | 0.8% | | 0.0% | 6.2% | | 99.2% | 0.8% | | 0.0% | 0.0% | 0.0% | | 60.8% | 0.0% | 93.8% | 0.0% | 7.7% |
|  | NA | 0.0% | 1.5% | 6.9% | | 6.2% | 0.0% | | 0.0% | 11.5% | | 6.9% | 0.0% | 0.0% | | 6.2% | 6.9% | 0.0% | 0.0% | 0.0% |
| **NVT** | S | 25.0% | 100% | 100% | | 100% | 100% | | 0.0% | 97.2% | | 100% | 100% | 100% | | 38.9% | 100% | 11.1% | 100% | 83.3% |
|  | I | 30.6% | 0.0% | 0.0% | | 0.0% | 0.0% | | 0.0% | 0.0% | | 0.0% | 0.0% | 0.0% | | 11.1% | 0.0% | 0.0% | 0.0% | 0.0% |
|  | R | 44.4% | 0.0% | 0.0% | | 0.0% | 0.0% | | 100.0% | 2.8% | | 0.0% | 0.0% | 0.0% | | 50.0% | 0.0% | 88.9% | 0.0% | 16.7% |
|  | NA | 0.0% | 0.0% | 0.0% | | 0.0% | 0.0% | | 0.0% | 0.0% | | 0.0% | 0.0% | 0.0% | | 0.0% | 0.0% | 0.0% | 0.0% | 0.0% |

**Table S3. Antimicrobial resistance genes of strains associated with serotype switches in *Streptococcus pneumoniae***

| **Index** | **ST** | **Serotype switch** | **genome number** | **Average AMR gene number** |
| --- | --- | --- | --- | --- |
| 1 | 320 | 19F-19A | 1 *vs.*7 | 10 *vs.*10 |
| 2 | 3173 | 6A-6B | 3 *vs*.2 | 8 *vs.*8 |
| 3 | 11972 | 15A-15B | 1 *vs.*2 | 8 *vs.*8 |
| 4 | 4749 | 14-15B | 2 *vs.*1 | 8 *vs* 8 |
| 5 | 2754 | 13-10B | 1 *vs.*2 | 8 *vs.*8 |
